# Italy’s contribution to global health: the need for a paradigm shift

**DOI:** 10.1186/1744-8603-10-25

**Published:** 2014-04-06

**Authors:** Eduardo Missoni, Fabrizio Tediosi, Guglielmo Pacileo, Lara Gautier

**Affiliations:** 1 Centre for Research on Health and Social Care Management (CERGAS), Università Bocconi, via Roentgen 1, 20136 Milano, Italy; 2 Swiss Tropical and Public Health Institute, P.O. Box, CH-4002, Basel, Switzerland; 3 University of Basel, P.O. Box, CH-4003, Basel, Switzerland

**Keywords:** Italy, Global health, Development assistance in health, Universal health coverage, Decentralized cooperation, Official development aid, Aid effectiveness, Italia, Salud Global, Ayuda al Desarrollo para la Salud, Cobertura Universal de Salud, Cooperación Descentralizada, Ayuda Pública al Desarrollo, Eficacia de la Ayuda

## Abstract

This paper reviews Italian Development Assistance for Health and overall contribution to Global Health from 2001 to 2012. It analyses strategies and roles of central and decentralized authorities as well as those of private non-profit and corporate actors. The research illustrates a very low and unstable official contribution that lags far behind internationally agreed upon objectives, a highly fragmented institutional scenario, and controversial political choices favouring “vertical” global initiatives undermining national health systems, and in contrast with Italian deep-rooted principles, traditional approaches and official guidelines.

Italy’s contribution to global health goes beyond official development aid, however. The raising movement toward Universal Health Coverage may offer an extraordinary opportunity for a leading role to a country whose National Health System is founded on the principles of universal and equitable access to care. At the same time, the distinctive experience of Italian decentralized cooperation, with the involvement of a multiplicity actors in a coordinated effort for cooperation in health with homologous partners in developing countries, may offer – if adequately harnessed - new opportunities for an Italian “system” of development cooperation. Nevertheless, the indispensable prerequisite of a substantial increase in public funding is challenged by the current economic crisis and domestic political situation. For a renewed Italian role in development and global health, a paradigm shift is needed, requiring both conceptual revision and deep institutional and managerial reforms to ensure an appropriate strategic direction and an efficient and effective use of resources.

## Abstract (es)

**Resumen:**

Este artículo analiza la contribución de Italia a la Salud Global y su Ayuda para el Desarrollo en Salud desde 2001 hasta 2012. Se analizan las estrategias y los roles de las autoridades centrales y descentralizadas, así como de los actores privados lucrativos y no lucrativos. La investigación muestra una contribución oficial muy baja e instable, muy por debajo de los objetivos internacionales acordados; un escenario institucional altamente fragmentado y líneas políticas controvertidas que han favorecido iniciativas globales “verticales” que por un lado socavan los sistemas nacionales de salud, y por el otro están en contradicción con arraigados principios, enfoques tradicionales y las líneas guías oficiales de Italia.

Pero la contribución de Italia a la salud global va más allá de la ayuda oficial al desarrollo. El creciente movimiento para la Cobertura Universal de Salud puede ofrecer una oportunidad extraordinaria de liderazgo para un país cuyo Sistema Nacional de Salud se funda en los principios del acceso universal y equitativo a la atención. Al mismo tiempo, si adecuadamente aprovechada, la peculiar experiencia italiana de cooperación descentralizada, que involucra una multiplicidad de actores en un esfuerzo coordinado de cooperación en salud con sus homólogos en los países en desarrollo puede ofrecer nuevas oportunidades par el “sistema” italiano de cooperación al desarrollo. Sin embargo, la actual crisis económica y la situación política nacional representan un desafío para el prerrequisito indispensable consistente en un substancial aumento de financiamientos públicos. Un rol renovado de Italia en temas de desarrollo y salud global, requiere un cambio paradigmático. Este necesita tanto un revisión conceptual, como profundas reformas institucionales y gerenciales, para asegurar una dirección estratégica apropiada y un uso eficiente y eficaz de los recursos.

## Background

Over the last two decades Italy’s quantitative record on Official Development Assistance (ODA) has been very poor and unstable. Its ODA/GNI (Gross National Income) ratio has been constantly less than 0.20% since 2000, which is substantially below the average of the Organisation for Economic Cooperation and Development (OECD) countries, members of the Development Assistance Committee (DAC). Peer reviews from the OECD have been critical regarding the management of Italian aid and the capacity of the public administration to overcome structural deficiencies [1,2]. The deepening of the economic crisis has contributed to a continuing downward trend, with substantial cuts in traditional sources of ODA^a^.

The overall weakness of the Italian Development Cooperation has inevitably affected the role the country could have played in fostering global health. In addition, Italy’s vision of international health cooperation suffered from other specific limiting factors.

Firstly, the decision to uncritically redirect Official Development Assistance in Health (DAH) to follow the push of more influential donors toward narrowly targeted, i.e. “vertical”, global initiatives, potentially undermining the effective delivery of integrated health care and the overall effectiveness of health systems [3], is inconsistent with Italy’s own official DAH guidelines and above all with its deep-rooted universalistic approach to health care. The Italian Constitution defines health as a “fundamental right of the individual and (an) interest of the community” (art. 32) and indicates “political, economic and social solidarity” as “intransgressible duties” (art. 2). The Italian National Health Service, (*Servizio Sanitario Nazionale - SSN*), founded in 1978, is financed by general taxation and, despite a certain degree of variability in the quality of its services across Italian regions, provides universal coverage and ensures a free choice of providers to patients through a pluralistic delivery structure (public and private), at relatively low cost [4,5].

Secondly, Italy has not adequately leveraged the various energies of its country system (“Sistema Italia”) [1,2] already involved in global health, including the experience of Italian institutions and civil society organizations in decentralized cooperation with homologous entities in partner countries. Partnerships for local human development have represented, since the early 1990s, a distinctive, although not unique feature of the Italian development cooperation experience [6,7].

Apart from institutional documents (largely in Italian) and some domestic reviews [8-10], internationally accessible literature on Italy’s global engagement in the health sector is limited in scope and outdated [11-13].

This article attempts to fill this gap. It first reviews principles and practice of the Italian approach to global health (and DAH). It then analyses relevant financial flows and trends, identifying main public and private actors involved in DAH, and exploring their contribution. We argue that there is a qualitative role for Italy to be plaid in the global health agenda, but economic and organizational challenges must be faced.

We conclude highlighting the need for a paradigm shift both in the overall Italian ODA governance, strategic direction and management, and in the way development cooperation is conceived.

## Methodology

The analysis is based on quantitative and qualitative information available on Italy’s engagement in global health. The overall observation period was limited to 2001–2012 since the beginning of the decade coincides with the “rapid-growth” period of DAH [14], as well as with the launch of, and Italian participation in selective, i.e. “vertical” global public-private partnerships.

We started by conducting a literature review through both Medline/Pubmed and Google Scholar without limiting the time period and variously combining the key word ‘Italy’ with ‘global health’, ‘international health,’ ‘Development Aid’, ‘Development Cooperation’ and ‘Development assistance’, and extending the search to studies written in both English and Italian.

Due to very limited findings of some relevance, we then extensively explored the websites and databases of relevant Italian Institutions and non governmental organizations including: the Ministry of Foreign Affairs (MFA), the Parliament, Regions, Interregional and Municipal Institutions, the Ministry of Health (MoH) and the National Health Institute – *Istituto Superiore di Sanità*, ISS, the National Council for Economy and Labour, - *Consiglio Nazionale dell’Economia e del Lavoro*, CNEL, the National Institute for Statistics, ISTAT, the Federations of Italian Development NGOs, and the Association of Banking Foundations (ACCRI). The search yielded official documents (including laws, regulations, guidelines, and reports) and pertinent quantitative data.

We then searched the websites of relevant International Institutions including the World Health Organization (WHO), the World Bank and the OECD, as well as global initiatives such as the Global Fund to Fight HIV/AIDS, Tuberculosis and Malaria (GFATM) and the GAVI Alliance. From these sites we also retrieved quantitative data concerning the Italian participation in WHO programs and global initiatives. Quantitative data related to ODA of the OECD-CRS (Creditor Reporting System) database were analysed to compare Italy’s commitments and their trends with those of other countries belonging to the G7 group and of the total DAC (without Italy). To this end we aggregated the two DAH related categories: Health (OECD-DAC code 120) and Populations Policies/Reproductive Health (OECD-DAC code130).

Information regarding global health advocacy and education was obtained through websites of leading civil society associations and networks such as the Italian Global Health Watch (*Osservatorio Italiano sulla Salute Globale* - OISG), the Italian Medical Students Secretariat, *Segretariato Italiano Studenti in Medicina* – SISM, the Italian Society of Migrations Medicine (*Società Italiana di Medicina delle Migrazioni* – SIMM) and the Italian Network for Global Health Education (*Rete Italiana per l’Insegnamento della Salute Globale* – RIISG).

From a quantitative standpoint the OECD CRS database does not allow a disaggregated analysis of sources of ODA below the national level. Italy lacks an integrated aid budget covering all the aid managed by the different government departments and by regional and local authorities [2]. This may lead to a substantial sub-estimation of the country’s ODA and overall DAH. Qualitative information equally suffers from the lack of a centralized comprehensive database, impeding to fully unveil the actual Italy’s contribution to global health.

## Results and discussion

### Italy’s Development Cooperation Institutional setting and DAH guidelines

Despite a clear need to tackle the structural deficiencies repeatedly highlighted in OECD’s Development Assistance Committee (DAC) peer reviews (in 2000, 2004 and 2009), several parliamentary attempts at reform have failed [1,2]. Italy’s foreign aid program is still carried out under the authority of Law No. 49 of 1987 [15], which places both the political direction and implementation of international development cooperation under the responsibility of the MFA and its Directorate General for Development Cooperation (Direzione Generale per la Cooperazione allo Sviluppo, DGCS). However, it is the Minister of Economy and Finance (MEF) that deals with the international financial institutions (IFIs) and with assessed contributions to multilateral agencies in development co-operation. In addition the MEF leads Italy’s participation in innovative global health financing mechanisms [2].

In addition to central authorities, Italian regions and autonomous provinces, have adopted laws to regulate decentralised international cooperation activities. Five regions (Emilia Romagna, Lombardia, Toscana, Umbria, Veneto) have also introduced specific regulations for their health development aid and established dedicated offices within their Regional Health Departments [16]. According to the law, municipalities and other local institutions, including local Health Authorities (*Aziende Sanitarie Locali, ASL*), are allowed allocating a limited portion of their annual budget to international cooperation initiatives [17]. Since the mid 1990s Decentralized Cooperation became distinctive feature of the Italian Cooperation with the objective of creating and consolidating long-term cultural, technical and economic partnerships between local communities as a tool for promoting human development. The partnership between local communities, the notion of territory (area of jurisdiction of a local authority), civil society seen as protagonist (cf. World Bank seeing it solely as beneficiary) and the promotion of consortia between local actors, represented a peculiarity of the Italian experience and were summarized in official guidelines [4,7].

In addition, Italy’s National Civil Protection department also participates in international relief operations, humanitarian aid, and “post-emergency” reconstruction projects (including access to water and health services) at the sites of natural disasters.

However, the lack of any mechanism to ensure policies are coherent with development objectives, make institutional co-ordination and monitoring difficult. In an attempt to respond to the challenge, in 2010, the MFA and the MEF jointly established an Inter-institutional Development Cooperation Board, open to representatives of central, regional and local public administrations, the corporate sector, academia, and NGOs. The vision is one of a “whole country approach” to development cooperation intended to reduce fragmentation and to build synergies among all the public and private stakeholders of the “System Italy” [18]. In practice, beyond good intentions to date the board has not produced any tangible result.

Regarding development cooperation in health, the twenty year-old DAH guiding principles [19] were revised in 2009 through a participatory process involving experts from a range of public and private institutions. Following the underlying concepts of the 1989 guidelines, which promoted both the Alma-Ata Declaration and a universalistic approach to health for all, the 2009 guidelines insisted on a system approach to health. Guidelines’ keywords are fight against poverty and socio-economic inequalities; universal and equitable access to health services; strengthening health systems; community participation; knowledge networks; and aid effectiveness for global health. It should be noted that in the guidelines the fight against infectious diseases (including HIV/AIDS, malaria and tuberculosis, and neglected diseases) is just one of the multiple objectives of equitable and universally accessible health systems [20]. Indeed, Italy abstained from purely vertical initiatives until the fight against HIV/AIDS became a central issue at the start of the new millennium. In the 2009 guidelines reference to global health initiatives, which Italy supported since 2001, is made only in the “political” preface [20]. The inconsistency between the Global Health guidelines based on expert advise, and the political-bureaucratic direction becomes highly evident in the Programming guidelines of the DGCS, where the focus is on the Italian participation to the GFATM, to innovative financial mechanisms such as the Advanced Market Commitment (AMC) and the International Finance facility for Immunisations (IFFIm), with only a side mention to strengthening health systems and universal health coverage (UHC) [18].

### The Italian Official Development Aid (ODA) flows

In 2012, Italy’s net ODA amounted to 0.13% of its Gross National Income (GNI), far below the UN and EU’s targets of 0.7% for EU15 member states by 2015 [21].

**Figure 1:**
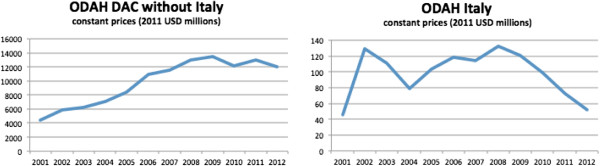
**ODAH trends.** DAC (without Italy) and Italy. Commitments in constant prices (2011 USD millions). Years 2001–2012. Source: authors elaboration on data extracted from OECD’s Creditor Reporting System on 18.12.2013.

**Figure 2:**
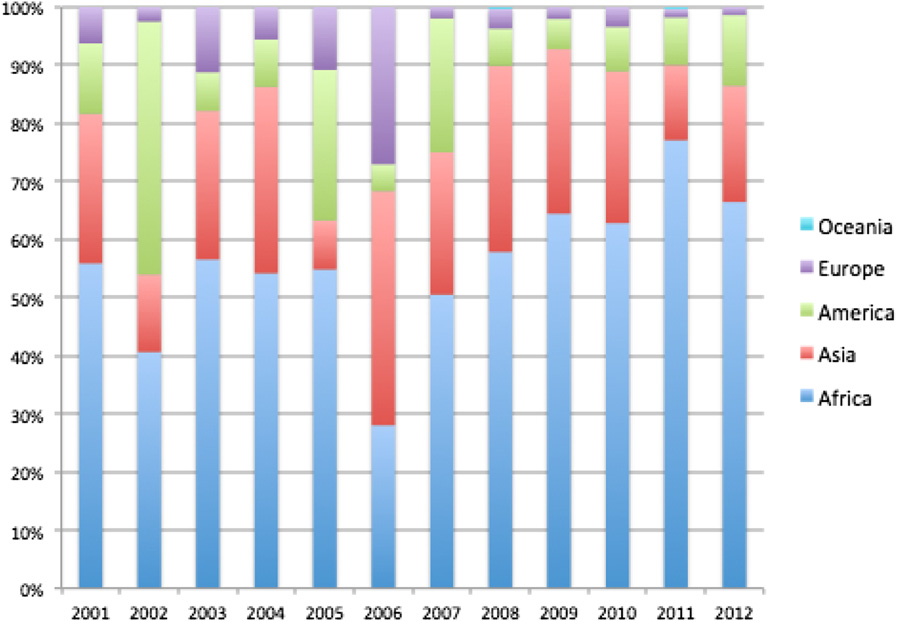
**Italy.** Geographically allocable ODAH. Distribution by Region. Years 2001–2012. Source: authors’ elaboration data extracted from OECD’s Creditor Reporting System on 18.12.2013.

Based on OECD’s CRS [22] Italy committed a total of US$ 1179 million from 2001–2012 (in 2011 constant prices) to official DAH (ODAH). The sharp decrease of Italy’s ODAH after 2008, contrasts with the relatively stable ODAH of the rest of the DAC (Figure 1). The share of traceable Italian ODAH channelled to recipient countries through multilateral organizations and Public-Private-Partnerships (data available only from 2006) also decreases after 2008, and a similar pattern is observed for ODAH channelled via NGOs and civil society. Over the same period ODAH’s share of the total sector allocable ODA fluctuated around an average of 14% (Table 1). Africa’s share of Italian ODAH grew substantially over the last five years and Sub-Saharan Africa remains a geographical priority for Italian ODA [18]. In 2011, Italian ODAH initiatives in Africa accounted for 70% of the geographically allocable funds for ODAH (Figure 2).

**Table 1:** Italian ODAH to all countries

	**2001**	**2002**	**2003**	**2004**	**2005**	**2006**	**2007**	**2008**	**2009**	**2010**	**2011**	**2012**
ODAH Total	45.8	129.7	111.0	79.0	104.0	118.7	114.8	132.6	120.6	99.5	72.0	52.0
Of which												
*via NGOs and Civil Society*	0.0	0.0	0.0	0.0	2.5	14.6	26.9	31.4	25.5	13.1	15.8	11.9
*Via Multilateral Organizations and PPPs*	0.0	0.0	0.0	0.0	0.0	17.2	10.0	18.1	7.5	5.4	6.0	6.8
*ODAH as % of total sector allocable*	73.9%	17.51%	14.97%	14.91%	11.58%	16.30%	16.86%	13.43	16.06	16.52%	14.39%	11.73%

Commitments at constant prices (2011 USD millions) Years 2001–2012.

Source: authors elaboration on data extracted from OECD’s Creditor Reporting System on 18.12.2013.

**Figure 3:**
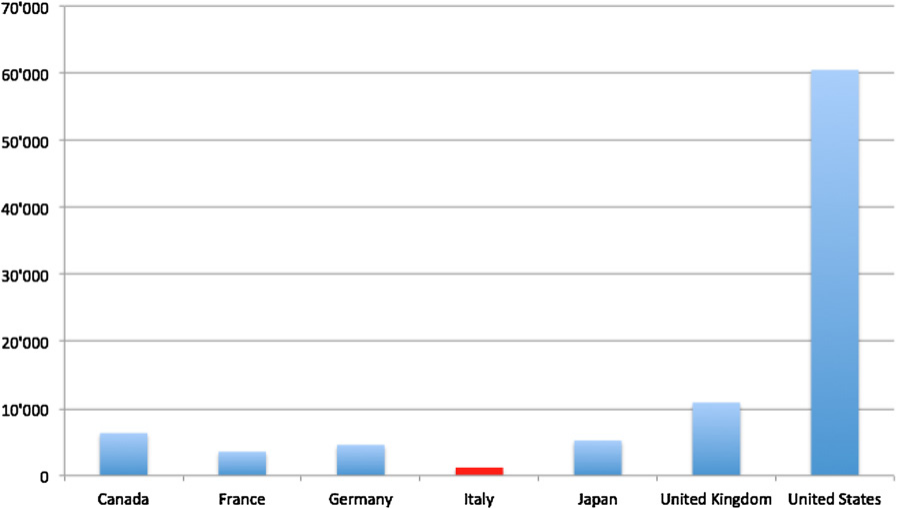
**G7 ODAH Commitments at constant prices (2011 USD millions).** Aggregated values 2001–2012. Source: authors’ elaboration on data extracted from OECD’s Creditor Reporting System on 18.12.2013.

**Figure 4:**
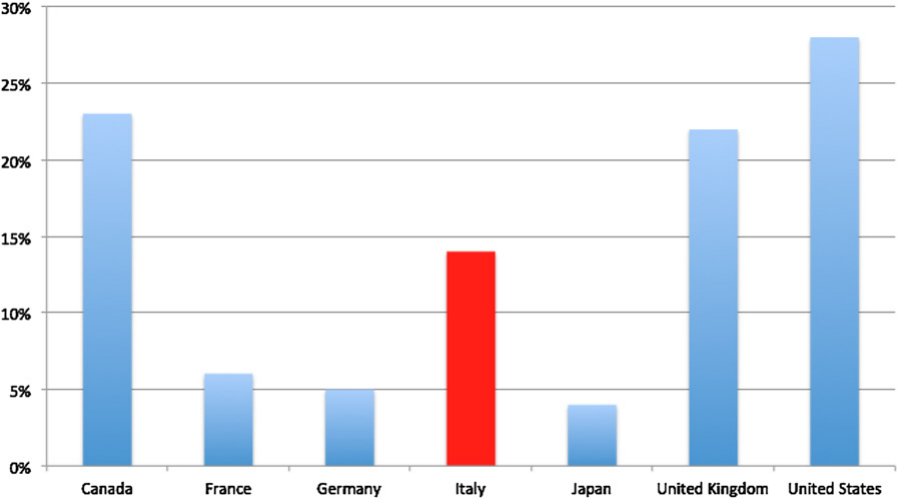
**G7 ODAH as percentage of total sector allocable ODA.** Aggregated values 2001–2012. Source: authors’ elaboration on data extracted from OECD’s Creditor Reporting System on 18.12.2013.

The same data series show that over the period 2001–2011 Italy’s ODAH lags far behind that of its fellow G7 countries in absolute terms (with less than half the ODAH of France which is the next worst performer) (Figure 3). However, looking at ODAH as a percentage of total sector allocable aid, Italy’s 14% ranks fourth behind the United States, Canada and the United Kingdom (Figure 4) [22].

### Italian DAH strategies and experience in global health

For years, Italy’s bilateral ODAH initiatives followed two main strategic lines: support to national and local health systems [23]. Local ownership, involvement of civil society, and coordination with all locally-relevant actors characterized the Italian approach to development, particularly in integrated human development and decentralized cooperation initiatives, which also strongly influenced a number of WHO [7] and UNDP programmes worldwide [24]. The participation to Sector Wide Approach (SWAp) programmes through budget support and other financial pooling mechanisms were laboriously initiated only in 2003 in Ethiopia and Uganda [23] and later extended to Mozambique, the Palestinian Territories, Niger, and Burkina Faso [25], and still face the challenging intricate aid budgeting and allocation procedures of Italian bureaucracy [2].

#### Support to global health initiatives

Besides supporting specific WHO-led initiatives through extra-budgetary contributions, Italy did not enter into global public-private partnerships until the GFATM’s launch at the Genoa G8 summit in 2001, which was itself the result of an “Tormented run-up” whereby economic actors heavily influenced the original agenda proposed by the Italian chaired G8 summit, which only a few months earlier was making explicit reference to the unaccomplished targets of Alma-Ata and recalled the ineffectiveness of selective approached to disease control [26]. Indeed, besides challenging traditional multilateral mechanisms, disease targeted initiatives sharply contrasted with Italy’s traditional “horizontal” and systemic approach to health. So much that even when deciding to put forward the proposal for a new global initiative, the Italian Presidency proposed a “Trust Fund for Health Care” [27] rather than a disease targeted facility which was finally launched by the G8.

Since its establishment in 2002, the GFATM became the most important beneficiary of Italian ODAH. With a total contribution of US$ 1008.3 million (2001–2009) Italy became GFATM’s eighth largest donor with a seat in GFATM’s board. However, Italy’s contribution has been unstable. For instance, in 2006 and in 2009, no contributions were made to the GFATM; Italy made up for this by honouring the pledges for 2008 in advance to regain credibility [8]. Since then, no further contributions were made [28], although in October 2012, former Italian Prime Minister Mario Monti ensured that Italy would not withdraw from its commitment to the GFATM, which was considered a “strategic investment” [29].

Other global health initiatives received Italian support, including: the Global Polio Eradication Initiative (US$ 39,76 million) [30]; Roll Back Malaria (over US$10 million) [31]; the Stop TB initiative (US$ 17.41 million from 2001–2010) [31]. In addition, Italy has pledged US$ 75 million over 2011–2015 to the Muskoka Initiative, but, to date, no contribution has been made [32,33].

Notwithstanding the concerns about supply-driven funding and earmarking of resources channelled through innovative financing mechanisms [34] and the contrast with its global health guidelines [20], Italy played a leading role in setting up the AMC, which aims to accelerate the development of new products by ensuring their subsequent purchase according to pre-arranged criteria with pharmaceutical companies [35]. With a pledge of US$ 645 million of the US$ 1.5 billion necessary for the development of a new pneumococcal vaccine, Italy ranked first among five donor countries [36]. Italy also pledged US$ 629.4 million (2006–2031) in support to the IFFIm, which raises funds on the capital market that the GAVI Alliance uses to purchase drugs and vaccines [37]. Overall, Italy’s total contribution to GAVI, through the IFFIm and the AMC, reached US$ 349.5 million from 2006–2011 [38].

#### The role of other public actors

The MoH and the ISS also undertake DAH interventions and have set up specialised offices for these activities.

The MoH is involved in many twinning projects with new EU-member countries, non-EU Mediterranean countries, and countries that were part of the former Soviet Union. These projects often involve the ISS, regional governments, research institutions, offices of the SSN, and healthcare providers [10]. Additionally, the MoH holds bilateral agreements on scientific cooperation, health information exchange, and health research with several developing countries, and set up a coordinating body for health cooperation initiatives with Mediterranean and Middle Eastern countries [39].

ISS is involved in DAH through the development of networks for promoting evidence-based medicine, health information systems, and training. Partner countries include China, South Africa, the Central Asian Republics, and multiple countries in Latin America, the Balkans, and the Middle East. ISS also provides humanitarian and technical assistance in collaboration with other international agencies [40].

In the past the SSN and its principles represented a model for some low- and middle-income countries pursuing a universalistic approach. This was the case of the Brazilian Unified Health System (Sistema Unico de Saúde, SUS) to whose development the Italian Development Cooperation contributed in the 1980’s [41].

Since the late 1990s, Italian regional governments have increasingly undertaken development cooperation activities, primarily in the health sector, which accounts for a large part of their budgets [16]. The regions’ DAH initiatives are implemented both directly through regional health services and indirectly through funding channelled by local and international NGOs. Activities include: humanitarian interventions; training and exchange programmes for health workers; heath promotion projects; provision of free healthcare in Italy to patients (mainly children) from developing countries. Italian regions also directly contribute to WHO programs (e.g. the Lombardia Region contributed more than US$600,000 to Stop TB [16]). Regional governments are also involved in (and co-fund) research programs of the EU and other international organisations as well as DAH initiatives initiated by the MFA, the MoH, and the ISS [40].

The total financial contribution of the regional and municipal governments to overall Italian DAH is difficult to estimate [2]. Information is only available for initiatives that have been entirely funded by regional governments, thus actual contributions can only be estimated. For instance, in 2007, the official expenditure on DAH of the five most important regions (Emilia Romagna, Lombardia, Toscana, Umbria, and Veneto) was estimated to be €8.5 million [16]. However, it may be assumed that this is only a fraction of the resources that the regions invest in DAH, considering extensive participation in initiatives that: a) involve partnerships with other national or international bodies, which are not included in the DAH budget, b) are implemented by local health authorities, ASL, or c) are implemented by other regional healthcare providers with independent budgets. Indeed, in the context of the Italian SSN, health is under the responsibility of Regions. In several cases these, as well as Municipalities, have been strong promoter of decentralized cooperation with the involvement of a multiplicity of institutional, academic and civil society actors in a coordinated effort for cooperation in health with homologous partners in DCs. Focus on co-development, cultural and technical interchange, reciprocity, and mutual accountability, and emphasis on Primary Health Care and integrated health systems are peculiar of the Italian decentralized cooperation approach and represent great potential for organizational innovation at territorial level. The experience of the Toscana region, and its recently instituted Global Health Centre, is a leading example [10].

#### Non-State actors

Other Italian actors play an increasingly relevant role in global health. According to the most recent data, there are 221,412 non-profit organizations in Italy, including 4,720 foundations. 1,433 of them are involved in international cooperation and solidarity activities, including health [42]. Non-profit organizations play an important advocacy role and have shown that they are capable of mobilizing sizeable resources from the private sector (in 2007, Italian non-profits raised approximately €341 million) [42]. Of these, 250 NGOs obtained accreditation as Development NGOs from the MFA [15,43]. This allows them accessing ODA funding to act as implementing agencies of governmental projects. In 2007, 104 accredited NGOs were implementing 507 projects in the health sector. Interestingly, 48.3% of these projects were funded entirely by private sources, 22.3% were either funded or co-funded by the MFA, and the rest were funded by other public national and international sources [44].

Italy is also home to many faith-based organizations that provide health services in low-income countries. Despite being independent and having international constituencies, they often rely on Italian personnel and refer to the Italian government and embassies for institutional support.

Large Italian corporate foundations and banking foundations have also displayed an increasing interest in being involved in global health issues. Banking foundations represent 88 non-profit entities that were constituted with the assets of saving banks dissolved in the 1990’s in accordance with specific legislation [45]. These foundations are already funding numerous domestic and international health projects and biomedical research. For example, in 2011 they disbursed €103.6 million for public health projects and €156.3 million for research [46]. A number of Italian banking and private foundations already started to explore innovative collaborative initiatives networking development NGOs, research centres and public institutions. Foundations could soon become major players in supporting Italian initiatives in global health.

The Italian corporate sector has also been showing an increasing interest in global health issues. For instance, the Italian Oil Company ENI and Giorgio Armani are among the few corporations that have contributed directly to the GFATM. In the countries where it has extraction activities (i.e. Azerbaijan, Congo, Libya, and Nigeria), ENI has been funding several health system development projects, as well as activities of international organizations such as UNICEF and WHO, through its corporate foundation [47].

#### Advocacy and global health education

Finally, since 2002, the Italian Global Health Watch (OISG) has been raising public awareness about global health. With OISG’s support, the Italian academic community introduced global health courses in medical schools (global health electives are now available in 26 medical schools), business schools, and faculties of social sciences, economics and management [48]. The Medical Students Association (SISM) and the Italian Society for Migrants Medicine (SIMM) are also significantly engaged nationwide in organizing global health courses. In March 2010, an informal consortium including all these actors- OISG, SISM, SIMM - the NGO, Doctors with Africa CUAMM, and a group of global health scholars, launched the Italian Network for Global Health Education (RIISG) [49]. This network has further contributed to the expansion of global health teaching and awareness in Italy. Health is a central issue in the wider academic debate on development cooperation, led by an *ad hoc* Coordinating body of academic institutions, the *Coordinamento Universitario per la Cooperazione allo Sviluppo* (CUCS) [50] Italian universities, both public and private, are establishing global health research units, such as the Center for International Health (*Centro di Salute Internazionale*) at the Public Health Institute at Bologna University (Bologna) [51], and the recently created Center for Global Health Research and Studies at the Catholic University (Rome) [52]. Despite this fertile environment, global health in academic institutions is sill a neglected area of research, embedded in the other traditional academic disciplines on which the Italian academia is still based. The lack of institutional attention and coordination, career and funding opportunities, is a major limitation, which contrasts with other European experiences where Development Cooperation agencies see their academic institutions as an important resource for their international mandate.

## Conclusions

Italy’s ODA financial performance has been poor for many years and the recent economic crisis is contributing to this downward trend with substantial cuts in traditional sources of Italian ODA. Waiting for a long overdue Reform, structural weakness of its governance and management structure further affects Italian ODA, which remains marked by fragmentation among various governmental bodies, lacking a clear political direction [2]. These weaknesses have been equally affecting the Italian DAH failing to harness the much stronger potentials of the Italian society. In fact, contradicting the principles rooted in the Constitution, those underpinning the 35 year old universalistic National Health Service, and diffuse awareness of the effectiveness of an integrated approach to health, Italy’s political leadership has adopted since 2001 a “me too” approach to DAH, passively following ideas and practices of arguable effectiveness pushed into the global agenda by few influential official and private donors.

To be relevant, Italy, which pledged to be among the major players in supporting the GFATM and a number of new financing mechanisms, should instead push for the harmonization of global initiatives and their alignment with beneficiaries’ management systems, in line with international recommendations for DAH effectiveness [34]. Harmonization and alignment should be the keywords also in catalysing domestic energies of “System Italy”.

Indeed, this would be in line with DAC recommendations [1] and consistent with the latest three year guidelines and planning directions (*Linee guida e indirizzi di programmazione*) of the DGCS which focus on the repeatedly announced “whole country approach” [1,18]. To that end, the involvement of the SSN and of decentralised public institutions, together with an extremely active civil society, the increasing relevance of private foundations, the growing social responsibility of the corporate sector, the commitment of academic institutions, and the privileged connection with the Catholic Church represent under-exploited cultural and operational resources.

Similarly the peculiarity of the Italian approach to decentralized cooperation should be fully harnessed, as well as its contribution to the overall debate on the essence of human development as a social process primarily aiming at satisfying population needs, and the conditions for its sustainability. Nevertheless, the potential of such an approach, rests in wide spaces of dialogue and interaction, and cannot be harnessed without a substantial increase in public funding and a structural reform of Italian ODA. Combining responsibility for all aid in a single Government department rather that having it split among various government departments; high level representation (at Cabinet level) with strong leadership skills and political profiles; wide consultation with outside experts; focus on long-term strategies centred primarily on improved life conditions in low-income countries and resistance to short-term pressures, including the promotion of national commercial interests, have been indicated as a combination of factors for excellence of a development agency, based on the experience of the British Department for International Development (DFID) [53]. Without adequate policy consistency, sound strategic direction and operational coordination, and a transparent and efficient administration of resources, a renewed Italian effort on the global development scene, to which the Italian contribution to global health is inevitably linked, will not be possible.

The potentials and energies that the Italian society can offer to the global health community are important and are currently undermined by country’s institutional and political weaknesses. The raise of Universal Health Coverage (UHC) on the global health agenda, despite ambiguities in the definition and little consensus in the concept [54], represents for Italy a great opportunity. Based on the consolidated experience of its universalistic national health service, Italy could play a prominent international role joining its voice, and sharing knowledge and experience with those partner countries that strive for attaining UHC and privilege equitable, comprehensive public systems. However, in the absence of a significant paradigmatic shift, along the above-mentioned conceptual, structural and operational lines, even changes in political and socioeconomic circumstances will not offer much hope.

## Endnote

^a^In 2012, reached a historic minimum of 0.13% of the GNI. The lowest among OECD-DAC countries.

## Abbreviations

ACCRI: Association of Italian banking foundations; AMC: Advanced market commitments; ASL: Aziende Sanitarie Locali (Local health authorities); CNEL: Consiglio Nazionale dell’Economia e del Lavoro (National council for economy and labour); CRS: Creditor reporting system; CUCS: Coordinamento Universitario per la Cooperazione allo Sviluppo (Coordination of universities for development cooperation); DAC: Development assistance committee; DAH: Development assistance in health; DFID: Department for international development; DGCS: Direzione Generale per la Cooperazione allo Sviluppo (Directorate General for Development Cooperation); EU: European union; GFATM: Global fund to fight HIV/AIDS, tuberculosis and malaria; GNI: Gross national income; IFFIm: International finance facility for immunisations; IFI: International financial institutions; ISS: Istituto Superiore di Sanità (National health institute); ISTAT: National institute fro statistics; MEF: Minister of economy and finance; MFA: Ministry of foreign affairs; MoH: Ministry of health; NGOs: Non governmental organizations; ODA: Official development assistance; ODAH: Official development assistance in health; OECD: Organisation for economic cooperation and development; OISG: Osservatorio Italiano sulla Salute Globale (Italian Global Health Watch); RIISG: Rete Italiana per l’Insegnamento della Salute Globale (Italian network for global health education); SIMM: Società Italiana di Medicina delle Migrazioni (Italian Society of Mirgrations Medicine); SISM: Segretariato Italiano Studenti in Medicina (Italian medical students Secretariat); SSN: Servizio Sanitario Nazionale (National health service); SUS: Sistema Unico de Saude (Unified health system); SWAp: Sector wide approach; UHC: Universal health coverage; WHO: World health organization.

## Competing interests

The authors declare that they have no competing interests.

## Authors’ contributions

EM, FT and GP conceived the original idea for the paper, designed the conceptual framework and equally contributed to the literature review and analysis of the results. LG contributed to the literature review and data collection, and provided comments and edits on various drafts. EM and FT wrote the manuscript. All authors have read and approved the final manuscript.
